# Lymph node dissection during nephroureterectomy: Establishing the existing evidence based on a review of the literature

**DOI:** 10.1080/2090598X.2019.1596401

**Published:** 2019-04-24

**Authors:** Nathan Grimes, Alastair McKay, Su-Min Lee, Omar M. Aboumarzouk

**Affiliations:** a Department of Urology, Monklands Hospital, Airdrie, UK; b Department of Urology, Glasgow Royal Infirmary, Glasgow, UK; c Department of Urology, Weston Area Health NHS Trust, Weston-super-Mare, UK; d Department of Urology, Queen Elizabeth University Hospital, Glasgow, UK; e University of Glasgow, Glasgow, UK

**Keywords:** Transitional cell carcinoma, nephroureterectomy, lymph node dissection

## Abstract

**Abstract Objective**: To determine the role of lymph node dissection (LND) in the treatment of upper tract transitional cell carcinoma (UTTCC), as the role of LND along with nephroureterectomy in treating UTTCC is unclear and several retrospective studies have been published on this topic with conflicting results.

**Methods**: The Medical Literature Analysis and Retrieval System Online (MEDLINE), the Excerpta Medica dataBASE (EMBASE), Cochrane Central Register of Controlled Trials database (CENTRAL), Cumulative Index to Nursing and Allied Health Literature (CINAHL), Clinicaltrials.gov, Google Scholar, and individual urological journals, were searched for all studies investigating the role of LND in the treatment of UTTCC. Of the studies identified, those that met inclusion criteria were included in this review.

**Results**: In all, 27 studies were included in this review, with 9303 patients who underwent LND. No randomised controlled trials (RCTs) were identified. Tumours were located in the renal pelvis in 62% of patients, in the ureter in 35.5%, and multifocal in 2.3%. In total: 77.1% were LN-negative and 22.9% had LN metastasis. For all patients undergoing LND, the 5-year recurrence-free survival (RFS) and cancer-specific survival (CSS) rates were 27–65.4% and 32.3–95%, respectively. For patients who underwent a LND in accordance with a standardised anatomical template, the 5-year RFS and CSS rates were 84.3–93% and 83.5–94%, respectively.

**Conclusion**: LND may provide a survival benefit in patients undergoing nephroureterectomy for UTTCC, particularly if following a standardised anatomical template and in those patients with muscle-invasive disease; however, a prospective RCT is required to confirm this.

**Abbreviations:** CSS: cancer-specific survival; LN(D): lymph node (dissection); MeSH: Medical Subject Headings; OS: overall survival; pT: pathological T stage; RCT: randomised controlled trial; RFS: recurrence-free survival; UTTCC: upper tract TCC

## Introduction

Urothelial carcinomas are the fifth commonest malignancy [1], of which 5–10% occur in the upper urinary tract [2,3]. They occur twice as commonly in the pelvicalyceal system as they do in the ureter [4]. In contrast with TCC of the bladder, where the majority of patients are diagnosed with non-muscle-invasive disease, about two-thirds of patients have muscle-invasive disease at diagnosis [5].

For patients with advanced disease, prognosis is poor. The 5-year survival is <50% in patients with pathological T stage (pT)2–3 disease and <10% in patients with pT4 disease [6–8]. Around 20–40% patients will have lymph node (LN) metastasis at diagnosis [9,10] and this is another strong predictor of poor prognosis, with 5-year survival rates of 10–30% [11,12]. In patients with locally advanced disease or with LN metastasis, adjuvant chemotherapy or radiotherapy rarely improves long-term survival [13–15].

Current guidelines by the European Association of Urology (EAU) advocate open or laparoscopic radical nephroureterectomy with bladder cuff excision as the standard treatment for high-risk upper tract urothelial cancer, regardless of tumour location [4]. Alternative treatments are generally reserved for patients with low-risk disease or those with significant renal impairment or solitary kidney.

The role of LN dissection (LND) for upper tract TCC (UTTCC) is not known and currently there are no guidelines regarding its role [4]. Given its rare nature, it is a difficult topic to establish an evidence base. In TCC of the bladder, there is increasing evidence that more extensive LND improves prognosis after radical cystectomy [16,17]. It may be the case that this same benefit may exist in treating UTTCC; however, the current evidence is based on small retrospective studies. Results from these studies has been conflicting, with some reporting a survival benefit of LND [9], whilst others report the only benefit being that of accurate staging for prognostication [18–20].

The aim of the present review was to systematically review the literature to establish the role of LND in patients undergoing nephroureterectomy for UTTCC.

## Methods

A review of the literature was conducted using Cochrane and Preferred Reporting Items for Systematic Reviews and Meta-Analyses (PRISMA) guidelines [21,22]. The search strategy included the following databases: The Medical Literature Analysis and Retrieval System Online (MEDLINE; 1980–2018), the Excerpta Medica dataBASE (EMBASE; 1980–2018), Cochrane Central Register of Controlled Trials (CENTRAL; in The Cochrane Library–2018), Cumulative Index to Nursing and Allied Health Literature (CINAHL; 1980–2018), Clinicaltrials.gov, Google Scholar, and individual urological journals.

Search terms used in conjunction with each other included: ‘upper tract urothelial neoplasms’, ‘lymph node’, ‘lymph’, ‘lymphadenectomy’, ‘lymph node excision’, ‘lymphatic’, and ‘nephroureterectomy’

Medical Subject Headings (MeSH) phrases included:

- ((“Lymph Nodes“[Mesh]) AND “Ureter”[Mesh]) AND ”Neoplasms”[Mesh])
- ((“Lymph Node Excision“[Mesh]) AND “Ureter”[Mesh]) AND ”Neoplasms”[Mesh])
- (((“Lymph Node Excision“[Mesh]) OR “Lymph Nodes“[Mesh]) OR ”Lymph”[Mesh]) AND ”Neph-rectomy”[Mesh]))

### Study selection

All languages were included if data were extractable, also references of searched papers were evaluated for further studies for potential inclusion. Authors were contacted wherever the data were not available or not clear, to be able to adequately assess inclusion of their study. If data were not extractable, provided or clarified, the study was excluded.

Inclusion criteria were: papers publishing outcome data for patients undergoing LND with nephroureterectomy for UTTCC, papers publishing original data (i.e., not review papers), and English language. Exclusion criteria were: abstracts published from conference proceedings with no full manuscript available, papers not providing outcome data specifically for patients who underwent LND at the time of nephroureterectomy, and papers publishing data not specifically for TCC.

### Data extraction

All types of publications were included. Studies were excluded if based on children or LN excision in other conditions than for UTTCC.

The following variables were extracted from each study: patient and cancer demographics, operation, LN yield, operative outcomes, and survival outcomes [recurrence-free survival (RFS), cancer-specific survival (CSS), and overall survival (OS)].

### Statistical analysis

We used the Review manager (RevMan) version 5.2 program (The Nordic Cochrane Centre, The Cochrane Collaboration, Copenhagen, Denmark) to conduct the analysis. For continuous data, a Mantel–Haenszel chi-squared test was used and expressed as the mean difference with 95% CI and for dichotomous data an inverse variance was used and expressed as risk ratio (RR) with 95% CI. A *P* < 0.05 was considered statistically significant [21,22].

Heterogeneity was analysed using a chi-squared test on N-1 degrees of freedom, with an α of 0.05 used for statistical significance and with the *I*^2^ test. *I*^2^ values of 0–40%, 30–60%, 50–90%, and 75–100% correspond to ‘heterogeneity may not be important’, ‘may indicate moderate heterogeneity’, ‘may indicate substantial heterogeneity’, and ‘may indicate considerable heterogeneity’, respectively [21,22]. A fixed-effect model was used unless statistically significantly high heterogeneity (*I*^2^ > 75% was considered as significantly high heterogeneity) existed between studies. A random-effects model was used if heterogeneity existed [22,23].

## Results

**Figure 1.**
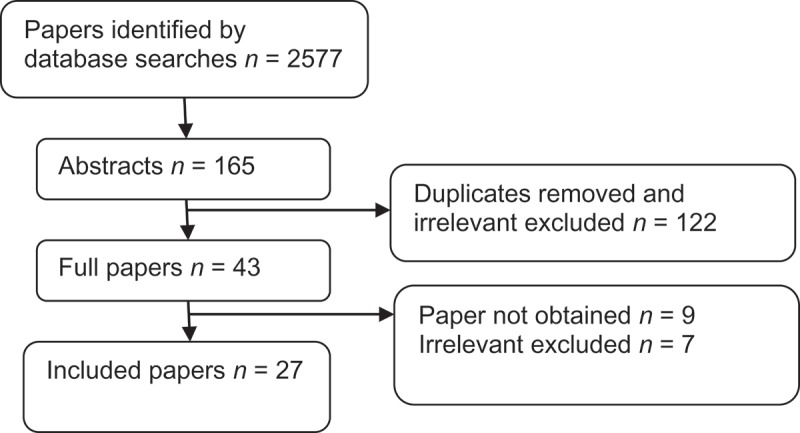
Inclusion of studies.

The initial database searches identified 2577 papers. Title review was conducted on all of these, and 2412 were deemed irrelevant and excluded leaving 165 papers. Abstracts were obtained and reviewed against inclusion and exclusion criteria; 122 papers were excluded at this stage leaving 43 papers. The full manuscripts of these remaining papers were obtained for final review against inclusion/exclusion criteria and for consideration of inclusion in the review. It was not possible to obtain nine of these papers via local or national resources or by direct contact with the authors. Of the 34 papers that were obtained in full, seven were excluded as they did not include survival data specifically for patients who had undergone LND. In all, 27 [24–50] were included in the present review, which are summarised in Table 1 [24–50] and methodology and search results are summarised in Figure 1.

**Table 1.** Study demographics.

Authors	Journal	Year	Data collection	Period included	No. of patients	Age range, years	Technique of nephroureterectomy (*n*)	Sex male/female, *n*	Disease location (*n*)	Histology (*n*)	Grade (*n*)	Stage (*n*)
Abe et al. [24]	*Eur J Surg Oncol*	2010	Retro.	1990–2005	293	38–90	Open (220) Laparoscopic (66) Open nephrectomy (7)	195/98	Pelvis (157) Ureter (112) Both (24)	TCC (267) Other (26)	G1/2 (185) G3 (108)	pTa/pTis (53) pT1 (66) pT2 (56) pT3 (101) pT4 (17)
Abe et al. [25]	*BJU Int*	2008	Retro.	1990–2005	312	38–90	Open (235) Laparoscopic (66)	207/105	Pelvis (169) Ureter (120) Both (23)	TCC (282) Other (30)	G1 (16) G2 (179) G3 (117)	pTa/pTis (54) pT1 (68) pT2 (59) pT3 (112) pT4 (19)
Bolenz et al. [26]	*BJU Int*	2008	Retro.	1992–2006	135	27–90	Open (119) Laparoscopic (16)	88/47	Pelvis (99) Ureter (36)	TCC	Low (10) High (125)	pT0 (1) pT1 (7) pT2 (21) pT3 (74) pT4 (32)
Brausi et al. [27]	*Eur Urol*	2007	Retro.	1980–2002	82	68 (median)	Nephroureterectomy (79) Partial nephrectomy (3) (disease in calyx of solitary kidney)	59/23	Pelvis (47) Ureter (28) Both (7)	TCC	G2 (44) G3 (38)	pT2 (38) pT3 (34) pT4 (8)
Burger et al. [28]	*World J Urol*	2011	Retro.	1987–2008	785	58–76	Open (715) Laparoscopic (70)	542/243		TCC	G1 (100) G2 (226) G3 (459)	pTa (165) pTis (10) pT1 (196) pT2 (148) pT3 (214) pT4 (43)
Cho et al. [29]	*J Korean Med Sci*	2009	Retro.	1986–2005	152	25–86	Open (152)	103/49	Pelvis (80) Ureter (72)	TCC	Low (14) High (138)	T2 (47) T3 (98) T4 (7)
Fujita et al. [30]	*Int J Urol*	2015	Retro.	1998–2013	74	50–81	Open (55) Laparoscopic (19)	48/26	Pelvis (37) Ureter (32) Both (5)	TCC	G1/2 (13) G3 (59) Unknown (2)	pTa–2 (12) pT3 (52) pT4 (10)
Furuse et al. [31]	*Jpn J Clin Oncol*	2017	Retro. prior to May 2010, prospect. thereafter	1994–2014	77	49–84	Open (49) Laparoscopic (28)	54/23	Pelvis (40) Ureter (37)	TCC	G1/2 (42) G3 (35)	pTis–1 (36) pT2–4 (41)
Ikeda et al. [32]	*Clin Genitourin Cancer*	2017	Retro.	1985–2013	399	IQR 61–76	Open (296) Laparoscopic (103)	307/92	Pelvis (213) Ureter (186)	Pure TCC (372) Other (27)	G1 (33) G2 (252) G3 (109)	pTa–1 (159) pT2 (78) pT3 (144) pT4 (18)
Inokuchi et al. [33]	*Jpn J Clin Oncol*	2017	Retro.	2005	823	IQR 61–77	Open (505) Laparoscopic (318)	578/245	Pelvis (434) Ureter (375) Both (14)	TCC (808) Other (15)	G1/2 (444) G3 (379)	pTa–1 (334) pT2 (155) pT3 (319) pT4 (15)
Komatsu et al [34].	*J Urol*	1997		1985–1993	36	41–84		21/15	Pelvis (19) Ureter (14) Both (3)	TCC	G1 (4) G2 (21) G3 (11)	pTa–1 (13) pT2 (5) pT3 (17) pT4 (1)
Kondo et al. [35]	*J Urol*	2007		1998–2006	169	38.7–85.5	Open (146) Laparoscopic (7) Nephrectomy (5) Segmental ureterectomy (7) Endoscopic ablation (4)	113/56	Pelvis (100) Ureter (69)	TCC		pTa–1 (45) pT2 (34) pT3 (79) pT4 (9)
Kondo et al. [36]	*Jpn J Clin Oncol*	2014	Retro.	1998–2013	180	36–91		128/52			Low (95) High (85)	pTa–1 (54) pT2 (25) pT3 (89) pT4 (12)
Kondo et al. [37]	*Int J Urol*	2014	Non-randomised prospect.	2005–2013	166	36–91	Open (108) Laparoscopic (58)	112/54	Pelvis (90) Ureter (76)			pTa–1 (62) pT2 (27) pT3 (72) pT4 (5)
Kondo et al. [38]	*Int J Urol*	2010	Retro.	1988–2009	119	38–90						pTa–1 (29) pT2 (22) pT3 (59) pT4 (9)
Kondo et al. [39]	*Int J Clin Oncol*	2017	Retro.	1988–2015	154	38–90		92/62	Ureter (154)	TCC	Low (37) High (117)	pTa–1 (50) pT2 (41) pT3 (62) pT4 (1)
Lughezzani et al. [40]	*Urology*	2010		1988–2004	2824	27–99		1666/1158	Pelvis (1913) Ureter (911)	TCC	G1 (156) G2 (935) G3 (1234) G4 (499)	pT1 (867) pT2 (500) pT3 (584) pT4 (873)
Mason et al. [41]	*Urology*	2012	Retro.	1990–2010	1029	Mean 68	Open (583) Laparoscopic (446)	654/375	Pelvis (538) Ureter (250) Both (213)		Low (340) High (689)	pT1 (463) T2 (160) T3 (244) T4 (54)
Ouzzane et al. [42]	*World J Urol*	2013	Retro.	1995–2010	714	IQR 61–76		484/229	Pelvis (388) Ureter (236) Both (90)		G1 (71) G2 (244) G3 (399)	pTa/is (209) pT1 (168) pT2 (74) pT3 (224) pT4 (39)
Roscigno et al. [43]	*J Urol*	2009	Retro.	1987–2007	1130	27–94	Open (924) Laparoscopic (206)				Low (291) High (839)	pT1 (317) pT2 (269) pT3–4 (544)
Roscigno et al. [44]	*Eur Urol*	2009	Retro.	1992–2006	421	27–94	Open (464) Laparoscopic (88)				Low (98) High (455)	pT1 (103) pT2 (114) pT3–4 (335)
Secin et al. [45]	*Int J Urol*	2007	Retro.	1985–2004	252	IQR 61–75	Open (248) Laparoscopic (7)	166/86				
Yoo et al. [46]	*Can Urol Assoc J*	2016	Retro.	2001–2013	287	Mean 66		196/91	Pelvis (105) Ureter (149) Both (33)		Low (84) High (203)	pTa–2 (146) pT3–4 (141)
Yoo et al. [47]	*World J Urol*	2017	Retro.	1998–2012	418	Mean 64	Open (184) Laparoscopic (234)	113/305	Pelvis/proximal ureter (206) Mid/distal ureter (153) Multifocal (59)		Low (200) High (218)	pTa–1 (205) pT2 (65) pT3-4 (148)
Youssef et al. [48]	*Int Braz J Urol*	2013	Retro.	1987–2007	22	52–89		15/7			High (22)	pT4 (22)
Youssef et al. [49]	*BJU Int*	2011	Retro.	1987–2007	313	27–97	Open (271) Laparoscopic (42)	109/204	Pelvis (1210) Ureter (95) Uretero-enteric anastomosis (8)		Low (45) High (268)	pT0 (6) pT1 (6) pT2 (83) pT3 (181) pT4 (37)
Zareba et al. [50]	*Cancer*	2017		2004–2012	14,472	IQR 61–78		8264/6208	Pelvis (9936) Ureter (4536)		Low (4628) High (8360)	pT0–1 (7627) pT2 (1873) pT3 (4183) pT4 (765)

IQR, interquartile range; Prospect., prospective; Retro., retrospective.

There were no randomised controlled trials (RCTs). In all, 21 studies [24–30,32,33,36,38,39,41–49] collected data retrospectively, one paper [37] collected data prospectively, and one paper [31] collected data both retrospectively and prospectively. In all, 17 papers included details of cohorts who had not undergone LND as a comparative cohort [24,27,28,31–33,35–37,39–43,45,47,50].

### Patient demographics

The 27 studies included a total of 25 969 patients, 9303 of whom underwent LND along with nephroureterectomy for UTTCC; patient characteristics are summarised in Table 1. Of those undergoing LND, 60.0% were male and ages ranged from 25 to 99 years.

In all, 23 papers [24–26,28,30–44,46,47,49,50] included patients regardless of pT stage, two papers included patients only with pT2–4 disease [27,29], and one paper only included patients with pT4 disease [48]. One paper [45] did not include data on pT stage of the patients included.

Two papers [35,48] did not provide specific data on pN stage of the patients included. Of the 25 papers that provided data on pN stage of patients, 18 [24–29,32,34,40–47,49,50] categorised patients into LN-positive or LN-negative, whilst the other seven [30,31,33,36–39] provided specific data on patients with each pN stage. LN status was provided for a total of 9131 patients: 77.1% were LN-negative and 22.9% had LN metastasis preoperatively.

Tumour location was documented in 7175 patients undergoing LND; 62.4% had a pelvic tumour, 35.5% had a ureteric tumour, and 2.3% had multifocal tumours. Three studies [24,32,33] included 35 patients who underwent LND for tumours other than pure TCC; however, this represented only 0.38% of all patients undergoing LND.

### Characteristics of included studies

In all, 12 studies presented data on RFS after LND [26,28,30–32,36,41–43,47–49]. Most of these papers reported RFS at 5 years from surgery. Eight studies provided 5-year RFS comparing patients who underwent LND (1792 patients) with patients who did not (3017) [28,32,36,39,41–43,47].

In all, 16 papers reported CSS [26,28,30–32,34,36,37,39–43,45,48,49]. Nine studies provided 5-year CSS comparing patients who underwent LND (3628 patients) with patients who did not (3597) [28,33,36,39–43,45].

Six papers reported OS after LND [33,37,41,42,49,50]. Three studies provided 5-year OS comparing patients who underwent LND (662 patients) with patients who did not (1499) [41,42,47].

### Surgical resection

Data published on patient treatment are summarised in Table 2 [24–50]. Details of surgical treatment generally applied to all patients included in the studies and not specified according to whether or not patients underwent LND.

**Table 2.** Interventions and outcomes.

Study	LND extent	Number of LNs removed	Adjuvant/neoadjuvant therapy	Complications	Perioperative mortality	Follow-up duration, months	OS	CSS	RFS
Abe et al. [24]	Discretion of surgeon	Median (range) of 6 (1–65)	30 patients had adjuvant chemotherapy in the setting of locally advanced disease (pT3–4 pNany) or LN-positive disease						Significant RFS in pN0 and pNx on multivariate analyses. Local recurrence HR 3.96 *P* = 0.003 Distant recurrence HR 2.86 *P* = 0.002
Abe et al. [25]	Discretion of surgeon	Median (range) of 6 (1–65)	36 patients had adjuvant chemotherapy in the setting of locally advanced disease (pT3–4 pNany) or LN-positive disease			Median (range) 47 (1–94)			
Bolenz et al. [26]	Discretion of surgeon		59 patients had adjuvant chemotherapy			Median (range) 49 (11–168)		5-year CSS 33% 10-year CSS 32%	5-year RFS 27% 10-year RFS 26%
Brausi et al. [27]	Discretion of surgeon	Before 1999 mean (range) 7.1 (5–10) After 1999 11.5 (5–24)			30-day mortality 0%	Median (range) 64.7 (24–288)		LND vs no LND 81.6% vs 44.8% *P* = 0.007	LND vs no LND 64.3% and 46.3% *P* = 0.03
Burger et al. [28]	Discretion of surgeon	Median (IQR) 3 (2–6)	69 patients had adjuvant chemotherapy			Median (IQR) 34 (15–65)		5-year CSS pN0 79% pNx 77.4% pN+ 26.7% *P* < 0.001 No statistically significant difference between pN0 and pNx	5-year RFS pN0 71.6% pNx 76.9% pN+ 21.3% *P* < 0.001; no significant difference difference between pN0 and pNx
Cho et al. [29]	Discretion of surgeon	Median (range) of 6 (1–25)	Disease infiltrating adipose tissue or LN disease 47 patients had adjuvant chemotherapy			Median (range) 53 (6–214)			No LND vs LND HR 3.46 *P* = 0.012
Fujita et al. [30]	Discretion of surgeon	Median (range) of 15 (12–45)	45 patients had adjuvant chemotherapy No criteria to decide who had adjuvant chemotherapy			Median (range) 20 (1–113)		Overall CSS at 2 years 54.6% and at 5 years 32.3%	Overall RFS at 2 years 37.9% and at 5 years 26.9%
Furuse et al. [31]	Before May 2010: discretion of surgeon. After May 2010: all LNDs were performed according to a specified template	SRLND median (range) 12 (3–34) Limited LND median (range) 3 (1–10)	3 patients in SRLND group had neoadjuvant chemotherapy 24 patients had adjuvant chemotherapy			Median (range): SRLND 46 (4–198) Limited LND 57 (17–169) No LND 72 (18–179)		SRLND vs no SRLND 94% vs 77% *P* = 0.015	5-year overall RFS SRLND vs no SRLND 93% vs 75% *P* = 0.005
Ikeda et al. [32]	Discretion of surgeon	Median (IQR) of 6 (3–10)	74 patients had adjuvant chemotherapy in the setting of pT3–4 or LN-positive disease			Median (IQR) 43 (17–89)		All patients: 5-year CSS *P* < 0.001 pN0 84.5% pNx 73.3% pN+ 43.6% pT2–4: 5-year CSS 77.1% vs 54.2% *P* = 0.001 pT3–4: 5-year CSS 76.3% vs 46.1% *P* = 0.001	All patients 5-year RFS *P* = 0.001 pN0 78.3% pNx 61.9% pN+ 33.2% pT2–4 pN0 vs pNx 5-year RFS 69.1% vs 43.1% *P* = 0.001 pT3–4 pN0 vs pNx 5-year RFS 66.4% vs 36.5% *P* = 0.001
Inokuchi et al. [33]	Discretion of surgeon		41patients had adjuvant chemotherapy in the LND group			Median (IQR) 59.8 (23.3–66.2)	pN+ OS 2-year 66% 5-year 50% No difference in survival in LND vs no LND No difference in survival in limited LND vs wider LND vs no LND		
Komatsu et al. [34]	Template dependent on tumour location		Adjuvant chemotherapy for patients with T3-4 or LN-positive disease 12 patients had adjuvant chemotherapy	No major complication or perioperative death	No major complication or perioperative death	Mean (range) 55 (3–135)		5-year CSS pN0 vs pN+ *P* < 0.001	
Kondo et al. [35]	Discretion of surgeon	Median of 7 Median (range) of 4 (2–30) for complete LND	LN disease or local infiltration of adipose tissue 12 patients had adjuvant chemotherapy in			Median (range) 37.3 (1–209)		All patients: no difference in CSS between complete/incomplete/no LND T3–4 patients: CSS better complete vs no LND at 5 years	
Kondo et al. [36]	Before December 2004 LND as per surgeon. After December 2004 LND as per template.	Median (range) of 3 (2–32) For incomplete LND Median of 11 for complete LND	9 patients had adjuvant chemotherapy			Median (range) Complete LND 47 (3–213) Incomplete LND 38 (7–208) No LND 26 (1–225)		2-year CSS Complete LND 95.1% Incomplete LND 83.7% No LND 82% 5-year CSS Complete LND 90.7% Incomplete LND 63.7% No LND 67.6%	2-year RFS Complete LND 87.4% Incomplete LND 80% No LND 71.3% 5-year RFS Complete LND 84.3% Incomplete LND 66% No LND 66.3%
Kondo et al. [37]	Standardised anatomical template	Median (range) of 4.5 (2–36)	Adjuvant chemotherapy was considered for patients with LN disease or infiltration of adipose tissue 10 patients in the LND group 2 patients in the No LND group	14.2% 90-day morbidity		Median of 4 cohorts 20–30 months, total range 1–103	Renal tumour: all patients OS HR 0.32; *P* = 0.02 Renal tumour T2–4 3-year OS 86.1% vs 48% *P* = 0.01	Renal tumour: all patients CSS HR 0.22 *P* = 0.01 Renal tumour T2-4 3-year CSS 89.8% vs 51.7% *P* = 0.01	Renal tumour: all patients RFS no difference Renal tumour T2–4 no difference in RFS
Kondo et al. [38]	LND as per surgeon prior to December 2004 LND as per template post January 2005	Median (range) of 9 (4–30) for complete LND and 4 (2–16) for incomplete LND	16 patients had adjuvant chemotherapy			Median (range) Complete LND 28 Incomplete LND 50 (1–221)		pT2–4: difference in survival for complete vs incomplete LND 5-year CSS *P* < 0.01	
Kondo et al. [39]	LND as per surgeon prior to December 2004 LND as per template post January 2005	Mean (range) of 13.5 (2–36) for complete LND and 4.5 (2–9) for incomplete LND	Adjuvant chemotherapy if positive LNs or tumour invasion into adipose tissue 16 patients had adjuvant chemotherapy			Mean (range) follow-up 33–88 (1–281)		2-year CSS *P* = 0.007 Complete LND 92.8% Incomplete LND 50% No LND 88.1% 5-year CSS *P* = 0.007 Complete LND 83.5% Incomplete LND 16.7% No LND 55.5%	pT2–4 of upper/middle ureter 2-year RFS *P* = 0.02 Complete LND 86.5% Incomplete LND 50% No LND 70.9% 5 year RFS p = 0.02 Complete LND 74.1% Incomplete LND 16.7% No LND 50.6%
Lughezzani et al. [40]						Median (range) 43 (0.1–203)		5-year CSS pNx vs pN0 no difference either over all (77.8% vs 81.2%) or T2–4 (71.3% vs 73.9%)	
Mason et al. [41]	Discretion of surgeon	Mean of 4.3	112 patients had adjuvant chemotherapy			Median (IQR) 19.8 (7.2–53.8)	5-year OS *P* < 0.01 pN0 66% pN+ 22.3% pNx 66.1%	5-year CSS *P* < 0.01 pN0 72.1% pN+ 29.8% pNx 74.7%	5-year RFS *P* < 0.01 pN0 39% pN+ 7% pNx 41%
Ouzzane et al. [42]	Discretion of surgeon	Median (IQR) of 2 (2–3)				Median (IQR) 27 (10–50)	5-year OS *P* = 0.001 pN0 68% pNx 74% pN1/2 40%	5-year CSS *P* < 0.001 pN0 81% pNx 85% pN1/2 47%	5-year RFS *P* = 0.001 pN0 66% pNx 77% pN1/2 42%
Roscigno et al. [43]	Discretion of surgeon		188 patients had adjuvant chemotherapy			Median (range) 45 (1–250)		Overall 5-year CSS 66% vs 69% *P* = 0.23 pN+ vs pNx vs pN0 5-year CSS 35% vs 69% vs 77% *P* < 0.001 and *P* = 0.032	Overall 5-year RFS 60% vs 65% *P* = 0.12 pN+ vs pNx vs pN0 5-year RFS 29% vs 66% vs 71% *P* < 0.001 and *P* = 0.045
Roscigno et al. [44]	Discretion of surgeon	Median (range) of 5 (1–41)	131 patients underwent adjuvant chemotherapy 34 patients received neoadjuvant chemotherapy			Median (range) 48 (1–246)			
Secin et al. [45]	Discretion of surgeon	Median (IQR) of 4 (2–10)	17 patients received adjuvant chemotherapy 7 patients received neoadjuvant chemotherapy 7 patients unclear if neoadjuvant or adjuvant This is overall, not necessarily LND			Median 37		3-year CSS *P* < 0.001 pN0 79% pNx 80% pN+ 41% 5-year CSS *P* < 0.001 pN0 56% pNx 73% pN+ 0%	
Yoo et al. [46]			Adjuvant chemotherapy if non-organ confined disease			Median (IQR) 38.4 (15.6–56.5)			RFS pN0 vs pN+ HR 6.8 *P* < 0.001
Yoo et al. [47]	LND depending on tumour location	Median (IQR) of 7 (3–10)				Mean 69	5-year OS LND vs no LND 72.1% vs 71.7% not statistically significant		5-year RFS, not statistically significant, not similar burden of disease between groups LND 65.4% No LND 76.4%
Youssef et al. [48]	Discretion of surgeon	Median (range) of 3 (0–20)	All had perioperative chemotherapy – unsure what proportion was neoadjuvant or adjuvant			Median 17		3-year CSS 28%	3-year RFS 35%
Youssef et al. [49]			18 patients had neoadjuvant and 88 had adjuvant Adjuvant chemotherapy if T3–4 or pN+					5-year CSS pN0–2 and chemo 44% pN+ 36% pN0 69%	5-year RFS pN0–2 and chemo 49% pN+ 30% pN0 64%
Zareba et al. [50]		Median (IQR) of 2 (1–6)	Overall 162 patients had neoadjuvant chemotherapy 1431 patients had adjuvant chemotherapy			Median (IQR) follow-up of survivors 42 (24–68)	5-year OS pN0 61% pN+ 24% pNx 59%		

HR, hazard ratio; IQR, interquartile range; SRLND, systematic regional LND.

In all, 18 studies [24–33,35,37,41,43–45,47,49] published data on the surgical procedures the patients underwent. Of these 7161 patients 99.6% underwent radical nephroureterectomy. In all, 18 patients underwent open nephrectomy [24,25], seven patients underwent segmental ureterectomy [35], three patients underwent partial nephrectomy [27], and four patients underwent endoscopic ablation [35]. Regarding the surgical technique, 74.8% of radical nephroureterectomies were performed open and 25.2% were performed laparoscopically.

In all, 16 studies [24–30,32,33,35,41–45,48] reported no set template for LND and extent was determined by the operating surgeon, two studies [34,37] reported an anatomical template for LND, and four studies [31,36,38,39] included patients with a mixture of LND performed at the discretion of the operating surgeon and following an anatomical template. It was not clear in five of the studies [40,46,47,49,50] whether or not LND was according to a pre-determined template.

### Adjuvant treatments

Use of adjuvant chemotherapy was reported in 23 studies [24–26,28–39,41,43–46,48–50]. As above, data were generally published on all patients included in each paper and not specifically on patients who underwent LND and were difficult to distinguish between the two groups. Indication for adjuvant chemotherapy was generally patients with pT3–4 disease or with LN metastasis. Five studies [31,44,45,49,50] reported the use of neoadjuvant chemotherapy. One paper [48] reported that patients underwent perioperative chemotherapy, but it was not clear if this was pre- or postoperatively.

### Perioperative morbidity and mortality (within 3 months of surgery)

Only three studies reported on these outcomes. Brausi et al. [27] reported a 0% 90-day mortality rate, Kondo et al. [37] reported a 14.2% 90-day morbidity rate, and Komatsu et al. [34] reported ‘no major complication or perioperative death’, i.e., 0%.

### Analysis results

#### RFS

**Figure 2.**
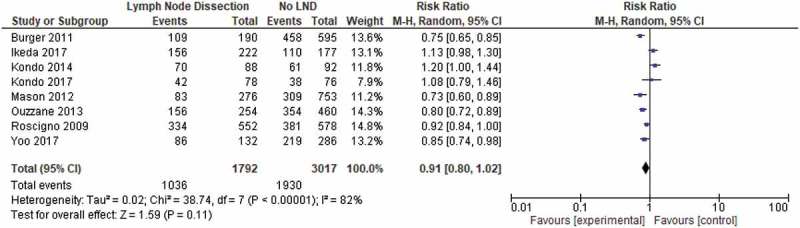
Pooled analysis of RFS.

The 5-year RFS ranged from 27% to 65.4%, whilst the 5-year survival was 39–71.6% for patients with pN0 disease and 7–37.9% for patients with LN-positive disease. Higher RFS was reported in patients when a specific template was followed for LND, 84.3–93% at 5 years [31,36]. One study reported 10-year RFS at 26% [26]. There was no significant difference in the 5-year RFS rate between those who underwent LND compared to those who did not (57.8% vs 64%; *P* = 0.11; Figure 2).

#### CSS

**Figure 3.**
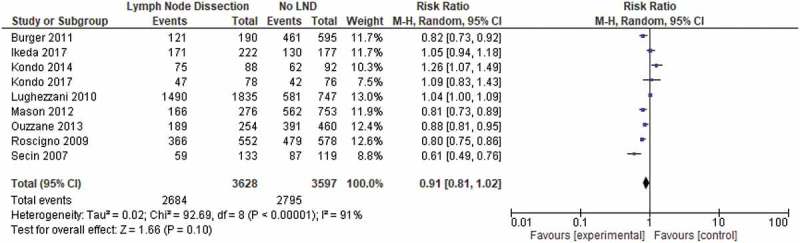
Pooled analysis of CSS.

The 5-year CSS ranged from 32.3% to 66%. One paper reported 10-year CSS at 32% [33]. Patients with pN0 disease had 5-year CSS of 56–84.5%, whilst patients with LN-positive disease had 5-year CSS ranging from 0% to 47%. Three studies published 5-year CSS for patients undergoing specific-template LND. The 5-year CSS was much better in these patients and ranged from 83.5% to 94% [31,36,39]. There was no significant difference in CSS between those who did and did not undergo LND, with a 5-year CSS of 74.0% and 80.5%, respectively (*P* = 0.1; Figure 3).

#### OS

**Figure 4.**
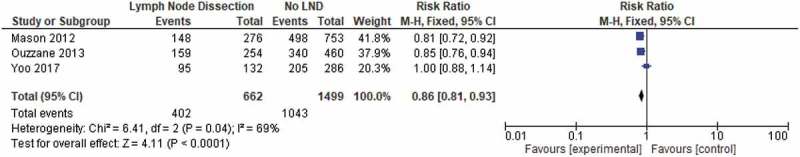
Pooled analysis of OS.

The 5-year OS for patients with pN0 disease ranged from 61% to 68% and was 22.3–66% for those with LN-positive disease. Kondo et al. [37] reported 3-year OS of 86.1% for patients with pT2–4 disease undergoing LND. The 5-year OS was 60.7% and 69.6% (*P* < 0.001) for those who did and did not undergo LND, respectively (Figure 4).

#### Impact on number of LNs removed or template

Six studies investigated the impact on number of LNs removed at LND [23,24,27,36,42,48]. One paper suggested there was no benefit in recurrence rates with increased LNs removed [26], whereas two papers reported reduced recurrence rates associated with increased number of LNs removed [29,44]. The number of LNs removed had a lesser impact on survival, with four papers reporting no difference in survival according to the number of LNs removed [25,26,38,44], whilst only one paper reported a small survival benefit with increased number of LNs removed [50].

Bolenz et al. [26] reported that increased number of LNs removed had no impact on either recurrence or survival, but did investigate proportion of positive LNs as a prognostic factor. Patients with >30% positive LNs had a higher rate of recurrence at 5 years compared with patients with <30% positive LNs (38% vs 25%, *P* = 0.021). Similarly, those with >30% positive LNs had a higher 5-year mortality rate (48% vs 30%, *P* = 0.032).

Five studies investigated whether a more standardised anatomical template for LND had an impact on outcomes [31,33,36,38,39]. Patients undergoing LND following a standardised template generally had better outcomes than patients undergoing LND not according to an anatomical template. Only one paper reported no difference according to type of LND [33]. Four papers [31,36,38,39] reported improved RFS after a standardised template and three papers reported improved survival when a standardised template was used for LND [31,36,38].

#### pN0 vs pNx

Five studies compared outcomes on patients who had undergone LND and been staged as pN0 with patients who were Nx [24,28,32,40,47]. One paper found patients with pN0 to have favourable RFS compared with patients who were Nx [24]. Two papers reported no difference in RFS between the two cohorts [40,47]. Burger et al. [28] reported that, overall, there was no difference in RFS between patients with pN0 and Nx staging, but when comparing patients with locally advanced disease, pN0 had improved RFS compared with those with Nx. Ikeda et al. [32] also reported better RFS in pN0 patients when only including patients with locally advanced disease. Four papers compared survival between pN0 and Nx patients [28,32,45,47]. Similarly to RFS, when all patients were included in analysis, there was no statistically significant difference noted between the two cohorts; however, when focusing on patients with locally advanced disease, two papers reported improved survival in patients who were pN0 [28,32].

## Discussion

There is a lack of high-quality evidence on the role of LND along with nephroureterectomy in treating patients with UTTCC. Furthermore, a vast disparity between countries and centres exists. It is not clear whether or not LND reduces recurrence or increases survival, or which patients may benefit most. However, patients who potentially benefit from LND are those with advanced disease and those who undergo LND according to a standardised anatomical template.

### Survival

Meta-analysis revealed no statistically significant difference in RFS and CSS between patients who did and did not undergo LND, but patients who underwent LND had poorer OS.

### LND

Another factor making it difficult to draw firm conclusions on the benefit of LND is the variation in what was included as LND in the different studies. Most of the studies simply stated that the extent of LND was at the discretion of the operating surgeon. In some cases, this resulted in as few as one LN being resected and in other cases as many as 65. Some studies performed LND in accordance with predetermined anatomical templates depending on the site of the primary tumour; these patients had much more favourable outcomes with 5-year CSS up to 94%.

### Chemotherapy

Comparison of outcomes between studies was further complicated by some patients undergoing neoadjuvant and/or adjuvant therapies in conjunction with nephroureterectomy. A large degree of variation was seen between the chemotherapy regimens described.

### Strengths and limitations of the review

The majority of published data are evidence based on retrospective studies with large degrees of heterogeneity between studies, and there were no RCTs. Nonetheless, the present review was conducted in a methodological protocol-driven method based on Cochrane and PRISMA guidelines.

The results presented represent the published existing data. Albeit, high risk of biases exist due to the heterogeneity between studies; however, this should emphasise the need for a multicentre RCT.

### Implications for clinical practice

Currently, with the existing studies from the literature, routine LND should not be advocated. If LND is required, a pre-determined templated technique should be used.

### Implications for research

It is clear that an RCT comparing between LND and no LND should be carried out to establish the evidence.

## Conclusion

There is currently insufficient evidence to support the role of LND along with nephroureterectomy in patients being treated for UTTCC. Some studies have suggested that patients with higher pT stage may be more likely to benefit from this and that a standardised anatomical template of LND results in better outcomes. A prospective, RCT is required to determine if there is a survival benefit in LND along with nephroureterectomy in patients being treated for UTTCC and in which group of patients, if at all, this is most appropriate.
